# Clinical, Laboratory, and Molecular Characteristics and Remission Status in Children With Severe Congenital and Non-congenital Neutropenia

**DOI:** 10.3389/fped.2018.00305

**Published:** 2018-10-16

**Authors:** Ruo-Lan Gong, Jing Wu, Tong-Xin Chen

**Affiliations:** Division of the Institute of Pediatric Translational Medicine, Department of Allergy and Immunology, Shanghai Childrens Medical Center, School of Medicine, Shanghai Jiao Tong University, Shanghai, China

**Keywords:** severe congenital neutropenia, severe idiopathic neutropenia, ELANE, G6PC3, primary immunodeficiency diseases

## Abstract

**Objectives:** Severe congenital neutropenia (SCN) is a primary immunodeficiency disease characterized by the early onset of recurrent infections and persistent severe neutropenia, with or without genetic defect. We aimed to study the different clinical features and hematological and bone marrow characteristics of patients with SCN and the non-congenital form of severe neutropenia (SN) with unknown etiology.

**Methods:** Thirty-nine Chinese children with severe neutropenia for longer than 6 months unrelated to virus infection or autoimmune diseases were enrolled in the study to analyse the clinical, laboratory, and molecular characteristics. They were followed clinically to observe their remission status.

**Results:** Seven patients were found to have SCN mutations, including *ELANE* and *G6PC3*. Among 26 patients with close follow-up, one died for an unknown reason, and 10 resolved spontaneously with a median neutropenia duration of 14.5 months; these patients were designated as having recovered SN. The demographic characteristics of both groups were similar, with a median infection rate of 5 times/year. SCN patients had more frequent infection than recovered SN patients (4 times/year, *P* = 0.039). The median absolute neutrophil count (ANC) was 0.40 × 10^9^/L in SCN patients, which was significantly higher than 0.2 × 10^9^/L in SN with unknown etiology and 0.21 × 10^9^/L in recovered SN patients (*P* = 0.021, *P* = 0.017). The median monocyte count was 1.60 × 10^9^/L in SCN patients, which was also significantly higher than 0.57 × 10^9^/L in SN of unknown etiology and 0.55 × 10^9^/L in recovered SN patients (*P* = 0.018, *P* = 0.001). Bone marrow examinations demonstrated myeloid maturation arrest at the myelocyte-metamyelocyte stage in SCN patients and normal findings in SN with unknown etiology and recovered SN patients.

**Conclusions:** Patients with severe neutropenia due to gene mutations demonstrate more serious symptoms than patients with unknown etiology. Patients with relatively higher ANC and monocyte counts are more likely to have known gene mutations. Future studies should focus on more detailed laboratory investigation, prolonged follow-up and advanced molecular biology tools to facilitate accurate diagnosis and effective treatment.

## Introduction

Primary immunodeficiency diseases (PID), of which more than 330 different disorders have been identified, are inherited disorders characterized by recurrent common or unusual microorganisms infections (1). Severe congenital neutropenia (SCN), a form of congenital phagocyte defect, is one of the most common types of PID (2).

As an inherited immunodeficiency disorder, SCN is characterized by persistent severe neutropenia (SN) and recurrent bacterial infection. Rolf Kostmann first reported cases of SCN in 1950 from a family with severe bacterial infections and neutropenia (3). SCN patients are typically characterized by severe neutropenia (< 0.5 × 10^9^/L), early onset recurrent bacterial infection, and predisposition to acute myeloid leukemia and myelodysplasia (4). The administration of recombinant human granulocyte colony-stimulating factor (G-CSF) as a treatment for severe bacterial infection may elevate a patient's absolute neutrophil count (ANC) to normal or close to normal range. Therefore, G-CSF can improve patients' quality of life (5).

With the advancement of DNA sequencing technology, SCN, a monogenic disorder, now has been confirmed to be caused by multiple genetic defects (6). Several studies indicated that heterozygous mutations of the neutrophil elastase gene (*ELANE*) may account for almost half of SCN cases (7–9). However, mutations in HS-1-associated protein X (*HAX1*) have also been verified to cause autosomal recessive SCN (10). Recently, mutations in *G6PC3* (G6PC3 deficiency), *GFI1* (GFI1 deficiency), *VPS45* (VPS45 deficiency), *G6PT1* (glycogen storage disease type 1b), *WAS* (X-linked neutropenia), *ROBLD3/LAMTOR2* (P14/LAMTOR2 deficiency), *TAZ* (Barth Syndrome), *COH1* (Cohen syndrome), *C16ORF57* (Clericuzio syndrome Poikiloderma with neutropenia), *JAGN1* (JAGN1 deficiency), *CLPB* (3-methylglutaconic aciduria), *CSF3R* (G-CSF receptor deficiency) were attributed to SCN (1).

In addition to SCN, there are other types of severe neutropenia, namely, autoimmune neutropenia (AIN) and severe idiopathic neutropenia (SIN) (11). AIN is associated with antibodies to human neutrophil antigens (HNA), including HNA-1, HNA-2, and HNA-3. However, the cause of severe neutropenia in those patients without autoimmune antibodies and genetic defects remains unknown. Since HNA testing is not routinely performed in most hospitals, the majority of SN cases still have an unknown etiology.

In the present study, we studied 39 cases of SN for which genetic analysis was conducted and analyzed clinical and laboratory findings in patients with or without a clear gene mutation. We also followed these patients to monitor their neutrophil counts and clinical progress. We identified unique differences between SCN and SN without etiology in terms of clinical features and hematological and bone marrow characteristics, aiming to improve the diagnosis and treatment of this disorder.

## Patients and methods

### Patients

This is a retrospective study of children from 5 to 132 months of age with severe neutropenia that was defined as ANC < 0.5 × 10^9^/L. A total of 665 patients were diagnosed with PIDs from June 2008 to July 2017 in hospitals affiliated to Shanghai Jiao Tong University School of Medicine. Among them, 39 patients (5.86%) were clinically diagnosed as having severe neutropenia with complete molecular analysis.

The criteria for conducting gene sequencing in such patients include: (1) severe neutropenia (ANC < 0.5 × 10^9^/L); (2) age of onset < 48 months; (3) duration of neutropenia > 6 months; (4) exclusion of active common virus infection (CMV, EBV, HIV, HHV6), no antinuclear antibodies (ANA), and no lympho-myeloproliferative disorders and myelodysplastic syndromes; (5) no use of anti-inflammatory medications within 3 months.

Patients with known gene mutations were diagnosed with SCN; otherwise, they were designated as having SN with unknown etiology. Twenty six of the 39 patients were followed until August 2017; 13 were deceased or lost to follow-up. Recovery of neutropenia was defined by our center as ANC > 1.0 × 10^9^/L for >3 months.

This study was approved by the Ethics Committee of Shanghai Children's Medical Center. Written informed consents were obtained from parents or legal guardians of patients.

### Data collection

Chart review was conducted to collect patients' clinical and laboratory data, such as origin, frequency, and types of infection, age at onset and diagnosis, family history, laboratory examinations, treatment methods, and outcomes. Clinical manifestations and hematological characteristics were followed every 6 months. Age of onset was defined as the age when ANC initially reached < 0.5 × 10^9^/L. Age of diagnosis was defined as the age when the diagnosis was first made by an immunologist based on gene testing. Patients with positive gene mutations were diagnosed as SCN, while others were designated as having SN with unknown etiology. The ANC values shown in Supplementary Table 2 reflected the time at which patients were clinically diagnosed without active infection and were not on therapy. ANC values shown in Supplementary Table 1 refers to patients' latest follow-up data. Bone marrow biopsy was performed as part of the workup for patients when other diagnoses needed to be excluded.

### Mutation analysis

Genomic DNA was extracted from peripheral blood samples of patients with ethylene diamine tetra acetic acid (EDTA) using a standard method. All coding exons and adjacent intronic regions of *ELANE, HAX1, G6PC3, GFI1, VPS45, G6PT1, WAS, ROBLD3/LAMTOR2, TAZ, COH1, C16ORF57, JAGN1, CLPB*, and *CSF3R* were amplified by polymerase chain reaction (1). The PCR products were then sequenced directly with second-generation technology. Sequence alignments were performed using the basic local alignment search tool (BLAST) from the National Center for Biotechnology Information (NCBI). New mutations were confirmed by screening 100 normal alleles from 50 unrelated, healthy Chinese individuals ranging from 18 to 40 years of age to rule out the possibility of polymorphisms in the Chinese population.

### Statistical analysis

Data are presented as the median, range, absolute number, or percentile. Statistical analyses were performed using SPSS 17.0 software. Comparison of median values between independent groups was carried out with the Mann–Whitney test. Differences between the groups were evaluated using the χ^2^ and Fisher exact tests. Correlation analysis was performed, and *P* < 0.05 was considered significant.

## Results

### Demographic information

A total of 39 clinical SN patients from 12 provinces of mainland China were enrolled. All of them fulfilled the diagnostic criteria mentioned above. The male-to-female ratio was 1:1.29. The median age at onset of symptoms was 4 months (0–36 months). The median age at time of diagnosis was 21 months (5–132 months, Table 1). The median diagnosis lag between onset of symptoms and medical visit was 12 months (3–132 months). A total of 74.36% (29/39) of the patients were diagnosed before the age of 36 months, and 3 patients were diagnosed after the age of 60 months (Table 1).

**Table 1:** Clinical characteristics in 39 Chinese patients with severe neutropenia.

**Pts**	**Year of diagnosis**	**Gender**	**Age(months)**				**Follow-up (years)**	**Family history**	**Infection**	**Infection frequency (times per year)**
			**Onset**	**Admission time**	**Admission lag**	**Status**				
P1	2008	M	24	54	30	162	11	–	Pneumonia, oral ulcer, suppurative otitis media, lymphadenitis	3
P2	2012	M	4	12	8	91	5	–	Pneumonia	2
P3	2012	M	0	132	132	210	5	–	Hepatic abscess, umbilical cord infection, pneumonia	4
P4	2013	F	2	10	8	60	4	–	Recurrent fever	5
P5	2013	M	8	14	6	64	4	–	Bronchitis, diarrhea	3
P6	2013	M	12	18	6	61	4	–	Recurrent fever	5
P7	2014	F	3	9	6	41	3	–	Pneumonia	3
P8	2014	F	1	25	24	56	3	–	Bronchitis, diarrhea	3
P9	2015	F	24	54	30	81	2	–	Pneumonia, purulent tonsillitis, rhinitis	8
P10	2015	M	1	7	6	33	2	–	Pneumonia	4
P11	2015	M	2	12	10	36	2	–	Purulent meningitis, Pneumonia	4
P12	2015	F	8	18	10	42	2	–	Recurrent fever	3
P13	2015	M	2	9	7	32	2	–	Pneumonia	3
P14	2015	F	6	38	32	61	2	–	Bronchitis	6
P15	2015	M	8	14	6	36	2	–	Pneumonia	5
P16	2015	F	24	48	24	67	2	–	Bronchitis	4
P17	2016	F	4	48	44	63	1	–	Bronchitis	4
P18	2016	F	19	33	14	48	1	–	Pneumonia	5
P19	2016	F	6	16	10	30	1	–	Scalp abscess	5
P20	2016	F	0	6	6	19	1	–	Purulent meningitis, septicemia, Pneumonia	4
P21	2016	F	27	108	81	120	1	–	Pneumonia	4
P22	2016	M	3	44	41	56	1	–	Bronchitis, Pneumonia	6
P23	2016	M	2	5	3	16	1	–	Pneumonia	4
P24	2016	F	36	88	52	99	1	+	Pneumonia	5
P25	2016	F	12	32	20	43	1	+	Pneumonia	5
P26	2016	F	0.	6	6	17	1	–	Diarrhea	2
P27	2016	M	4	8	4	Dead 9	1	–	Pneumonia, septicemia	4
P28	2016	M	6	51	45	62	1	–	Recurrent fever	5
P29	2016	F	15	21	6	30	1	–	Pneumonia, diarrhea	4
P30	2016	F	14	21	7	29	1	–	Pneumonia	3
P31	2016	M	1	7	6	15	1	–	hematochezia, bronchitis	5
P32	2016	F	3	26	23	33	1		Pneumonia	4
P33	2016	F	6	24	18	31	1	–	Pneumonia	3
P34	2016	M	1	32	31	39	1	–	Pneumonia, oral ulcer, suppurative otitis media, purulent parotitis	8
P35	2017	F	5	24	19	30	0.5	–	Pneumonia	3
P36	2017	F	8	16	8	21	0.5	–	Pneumonia	5
P37	2017	F	1	21	20	25	0.3	–	Pneumonia	2
P38	2017	M	2	14	12	16	0.2	–	Pneumonia, appendiceal abscess	6
P39	2017	M	1	14	13	16	0.2	–	Pneumonia, recurrent fever, scalp abscess	5

**Figure 1:**
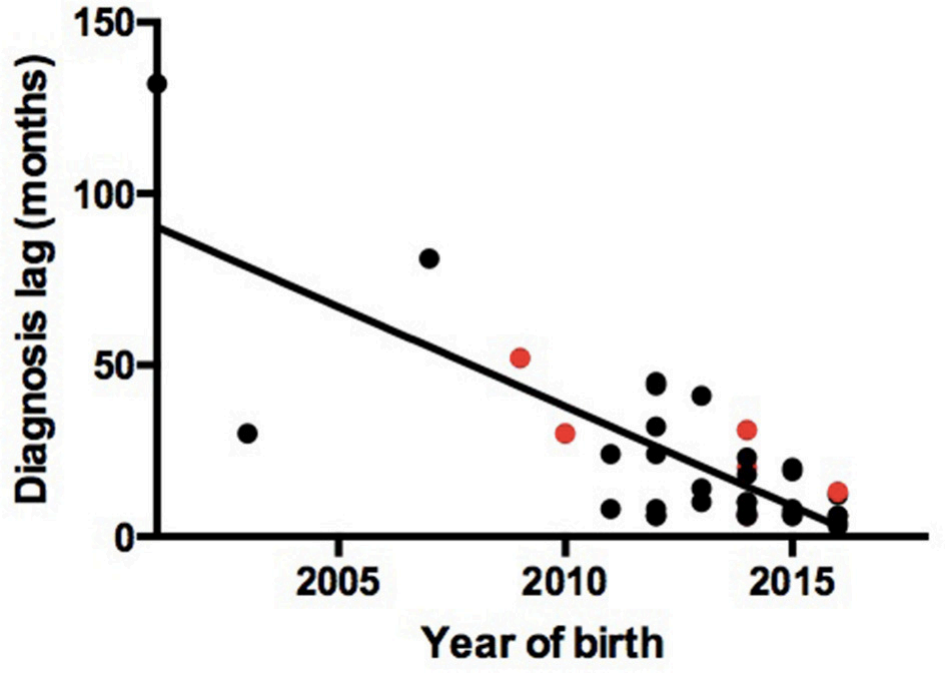
Association between year of birth and diagnosis lag. Red points represent patients with gene mutations. Black points represent patients without gene mutations.

We observed a shorter lag in diagnosis with more recent patients (*r* = −0.78, *F* = 55.85, *P* < 0.0001), indicating increased awareness and advances in diagnostic technology (Figure 1). The 39 patients were from 38 unrelated, non-consanguineous families. P24 and P25 were siblings whose father, not included in our study, had the same symptoms. The remaining 37 patients had no positive family history (Table 1).

### Molecular characteristics

Molecular analysis was conducted in all 39 patients (Table 2). All published SCN gene mutations, such as *ELANE, HAX-1*, and *G6PC3*, and all other reported PID mutations were sequenced in these patients. Only 7 (17.95%, 7/39) patients had detectable gene mutations (Table 2). With the exception of P34, with a compound heterozygous mutation of *G6PC3*, the other 6 patients carried heterozygous mutations in *ELANE*. P9 and P10, from two unrelated families, both had the missense mutation c.377C>T (p.S126L). P38 and P39 had the same heterozygous nonsense mutation, c.640G>A (p.G214R). P24 and her sister P25 were heterozygotes for a nonsense mutation of *ELANE*, c.580C>T (p.Q194ter), which was inherited from their father. All *ELANE* mutations were confirmed to be pathogenic mutations (12). P34 was the only compound heterozygote for *G6PC3* mutations (c.295C>T/p.Q99X from his father, c.766_768del/p.256_256del from his mother). Mutations in *G6PC3* were novel mutations that were not found in 100 normal alleles of 50 unrelated, healthy individuals. As a result, P9, P10, P24, P25, P34, P38, and P39 were diagnosed as SCN. However, the other patients were classified as having SN with unknown etiology.

**Table 2:** Molecular characteristics of patients with SCN.

**Pts**	**Gene**	**cDNA**	**Protein**	**Mutation type**
P9	ELANE	c.377C>T	p.S126L	Missense
P10	ELANE	c.377C>T	p.S126L	Missense
P24	ELANE	c.580C>T	p.Q194Ter	Nonsense
P25	ELANE	c.580C>T	p.Q194Ter	Nonsense
P34	G6PC3	c.295C>T c.766_768del	p.Q99X p.256_256del	Nonsense deletion
P38	ELANE	c.640G>A	p.G214R	Nonsense
P39	ELANE	c.640G>A	p. G214R	Nonsense

### Follow-up and outcome

The mean follow-up period for all patients was 1.92 years. Two-thirds of the patients (26/39, including 7 SCN and 19 SN with unknown etiology) completed follow-up, while the rest (33.33%, 13/39) were lost to follow-up. P27 died at 9 months of age from severe sepsis. All 39 patients received irregular G-CSF treatment (unknown dose), and 8 patients (P3, P10, P14, P16, P21, P23, P27, P39) received IVIG treatment. None of them developed malignancy.

**Figure 2:**
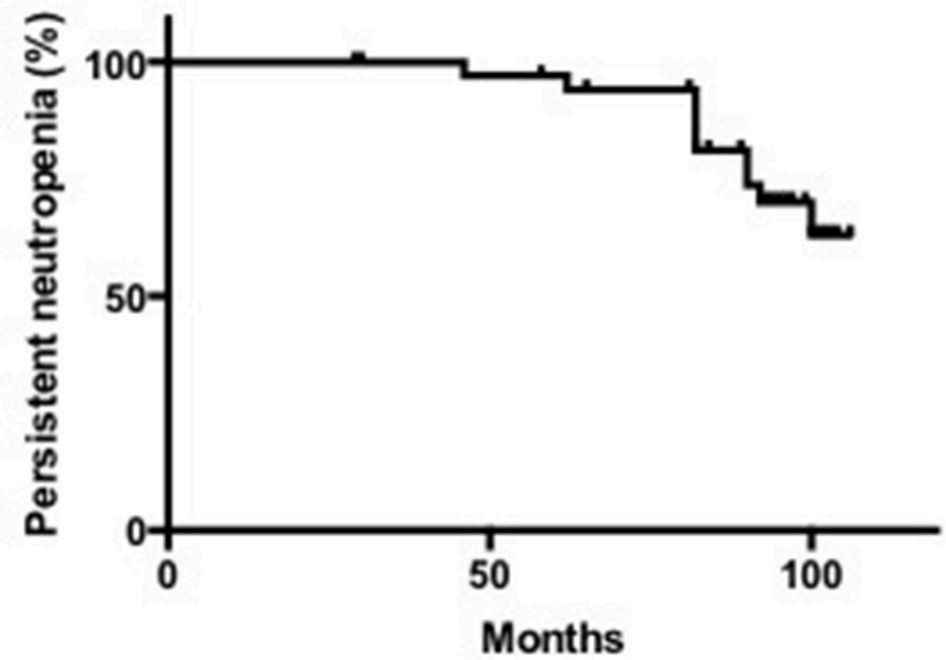
Remission rate of neutropenia in patients with SN.

The median last ANC count was 0.70 × 10^9^/L, with a range of 0.02–2.30 × 10^9^/L. According to the criteria for neutropenia recovery that we defined above, 52.63% (10/19) of SN patients with unknown etiology recovered from neutropenia. The median age of recovery was 23.5 months, ranging from 11 to 41 months. The median time to recovery was 12 months, ranging from 3 to 19 months (Supplementary Table 1, Figure 2).

The median duration of neutropenia in the 26 patients was 25 months, ranging from 10 to 138 months. Among them, the median time needed for neutropenia recovery (stable neutrophils >30 months) was 14.5 months, ranging from 10 to 39 months. For patients without neutropenia recovery, the neutropenia duration was 31.5 months, ranging from 13 to 138 months (Supplementary Table 1). Based on above data, we divided these patients into 3 groups: SCN, SN with unknown etiology, and recovered SN patients.

### Subgroup information

The male-to-female ratio was 1:0.75 in the SCN group, 1:3.5 in the SN with unknown etiology group, and 1:2.33 in the recovered SN group. The median age at onset of symptoms was 2 months (1–36) in the SCN group, 6 months (1–24) in the SN with unknown etiology group, and 3 months (0–19) in the recovered SN group. The median age at diagnosis was 32 months (7–88) in the SCN group, 24 months (16–54) in the SN with unknown etiology group, and 16 months (5–38) in the recovered SN group. The median diagnosis lag was 20 months (6–52) in the SCN group, 20 months (8–44) in the SN with unknown etiology group, and 8.5 months (3–32) in the recovered SN group. All of the above data did not show significant differences among the SCN, SN with unknown etiology and recovered SN groups (all *P* > 0.05) (Table 3).

**Table 3:** Demographic data, clinical features, and hematological characteristics of patients with SCN, SN with unknown etiology and recovered SN.

	**SCN**	**SN with unknown etiology**	**Recovered SN**	***P*****-value**		
				**Between SCN and SN with unknown etiology**	**Between SCN and recovered SN**	**Between SN with unknown etiology and recovered SN**
**GROUPING DEMOGRAPHIC DATA**						
Male to female ratios	1: 0.75	1: 3.5	1: 2.33	*P* > 0.05	*P* > 0.05	*P* > 0.05
Median age at onset of symptoms (months)	2	6	3	*P* > 0.05	*P* > 0.05	*P* > 0.05
	Range 1–36	Range 1–24	Range 0–19			
Median age at diagnosis time (months)	32	24	16	*P* > 0.05	*P* > 0.05	*P* > 0.05
	Range 7–88	Range 16-54	Range 5–38			
Median diagnosis lag (months)	20	20	8.5	*P* > 0.05	*P* > 0.05	*P* > 0.05
	Range 6–52	Range 8–44	Range 3–32			
**CLINICAL FEATURES**						
Median types of infection	2	1	1	*P* > 0.05	*P* > 0.05	*P* > 0.05
	Range 1–4	Range 1–4	Range 1–4			
Frequency of infections (times/per year)	5	4	4	*P* > 0.05	*P* = 0.039	*P* > 0.05
	Range 4–8	2–6 times	Range 2–6 times			
**HEMATOLOGICAL CHARACTERISTICS**						
Median count of ANC (× 10^9^/L)	0.40	0.2	0.21	*P* = 0.021	*P* = 0.017	*P* > 0.05
	Range 0.05–0.50	Range 0.02–0.47	Range 0.01–0.50			
Median count of monocytes (× 10^9^/L)	1.60	0.57	0.55	*P* = 0.018	*P* = 0.001	*P* > 0.05
	Range 0.80–2.44	Range 0.40–1.27	Range 0.00–1.40			
Median count of WBC (× 10^9^/L)^*^	5.34	4.15	4.40	*P* > 0.05	*P* > 0.05	*P* > 0.05
	Range 3.67–8.70	Range 1.60–6.78	Range 1.11–8.86			

* *Although the result was not significant enough, it could also be seen a little a higher tendency of WBC count in SCN group compared to that in SN with unknown etiology and recovered SN group (P = 0.07, P = 0.12)*.

### Clinical features

**Figure 3:**
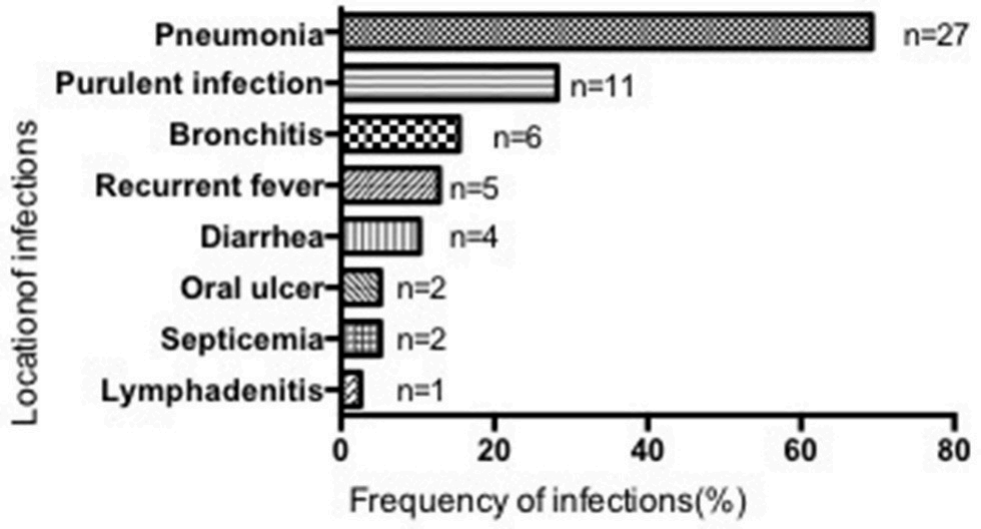
Location and frequency of infections in all patients.

Most patients suffered from respiratory infections, with pneumonia being the most common (69.23%, 27/39 cases), followed by purulent (28.21%, 11/39), cutaneous (2 cases), otic (2 cases), meningeal (2 cases), appendiceal (1 case), parotitic (1 case), hepatic (1 case), tonsillar (1 case), and umbilical infections (1 case). No hepatosplenomegaly was observed (Figure 3). The median number of infection types was 2 (1–4) in the SCN group, 1 (1–4) in the SN with unknown etiology group, and 1 (1–4) in the recovered SN group. There was no difference between these 3 groups (*P* > 0.05) (Table 3).

Although all patients had a history of recurrent infections, the frequency of infections varied among patients with a median of 5 times/year (4–8) in the SCN group, 4/year (2–6) in the SN with unknown etiology group, and 4/year (2–6) in the recovered SN group. The SCN group had significantly more infections than the recovered SN group (*P* = 0.039), while there was no difference between the other two groups (*P* > 0.05) (Table 3).

### Hematological characteristics

All patients had ANC values < 0.5 × 10^9^/L. The median ANC count was 0.40 × 10^9^/L (0.05–0.50) in the SCN group, 0.2 × 10^9^/L (0.02–0.47) in the SN with unknown etiology group, and 0.21 × 10^9^/L (0.01–0.50) in the recovered SN group. The SCN group showed significantly higher ANC values than those of the SN with unknown etiology and recovered SN groups (*P* = 0.021, *P* = 0.017). Nevertheless, the median ANC showed no difference between the SN with unknown etiology and recovered SN groups (*P* > 0.05, Table 3, Supplementary Table 2).

The median monocyte count was 1.60 × 10^9^/L (0.80–2.44) in the SCN group, 0.57 × 10^9^/L (0.40–1.27) in the SN with unknown etiology SN group, and 0.55 × 10^9^/L (0.00–1.40) in the recovered SN group. Monocyte counts of the SCN group was significantly higher than those of the SN with unknown etiology and recovered SN groups (*P* = 0.018, *P* = 0.001). However, no difference was observed between the latter two groups (*P* > 0.05, Table 3, Supplementary Table 2).

The white blood cell (WBC) count was 5.34 × 10^9^/L (3.67–8.70) in the SCN group, 4.15 × 10^9^/L (1.60–6.78) in the SN with unknown etiology group, and 4.40 × 10^9^/L (1.11–8.86) in the recovered SN group and was not statistically significant between groups (*P* = 0.07, *P* = 0.12). There was no difference between the SN with unknown etiology and recovered SN groups (*P* > 0.05, Table 3, Supplementary Table 2). Other hematological characteristics also showed no significant differences among the 3 groups (data not shown).

### Immunological characteristics

With the exclusion of patients who received intravaenous immunoglobulin within 3 months, the median IgG, IgM, and IgA levels were all within normal ranges (data not shown). There were no significant differences among the 3 groups in terms of IgG, IgM, and IgA levels (*P*-values were all >0.05, data not shown).

### Bone marrow characteristics

Bone marrow biopsy in SCN patients demonstrated a mild maturation arrest of granulocytes at the myelocyte-metamyelocyte stage with toxic granulation, vacuolar degeneration, and a marked absence of mature granulocytes. Nucleated cells, erythroid cells, and megakaryocytes were normal in number and morphology. With the exception of P16, P21, and P27, other SN with unknown etiology and recovered SN patients had normal bone marrow biopsy results. The results of P16, P21, and P27 were similar to those of SCN patients (data not shown).

## Discussion

We reported the demographics, molecular features, and remission status of 39 patients, classified as having SCN, SN with unknown etiology and recovered SN. We compared the clinical, immuno-hematological characteristics and bone marrow biopsy results in these 3 groups. Due to the lack of accurate available tests for measuring antineutrophil antibodies, a diagnosis of autoimmune neutropenia could not be given in this study. Therefore, a correlation between gene mutations and autoimmune neutropenia could not be made. However, we still found several unique distinctions between patients with SCN and SN without known etiology, which may help physicians differentiate types of neutropenia before gene analysis.

No gender difference was noticed among the different groups of patients. This is consistent with the literature showing that patients with SCN and SN with unknown etiology encompassed equal numbers of males and females (12, 13). The median diagnosis lag was 12 months, which is similar to the lag of 15 months found in Rezaei's study (14). Neutropenia can be accessed without the sophisticated instruments needed for other immunodeficiency diseases. With improved access to complete blood cell counts, we also found that the diagnosis lag decreased significantly in the last few years. With the exception of two sisters diagnosed as SCN, no other patients were related to each other biologically. Two thirds of the patients (66.67%) first manifested neutropenia during the first 6 months of life. It is recognized that early onset of recurrent bacterial infections (within the first 6 months) is one of the characteristics of SCN patients (4).

To date, more than 10 gene mutations have been reported to cause SCN, such as *ELANE, HAX1*, and *G6PC3* (4, 13). SCN is genetically heterogeneous. However, over 60% of autosomal dominant and sporadic cases of SCN are associated with mutations of *ELANE. ELANE* encodes neutrophil elastase, and its mutation increases the susceptibility of neutrophils to apoptosis through initiation of the unfolded protein response (9, 15). Mutations in *G6PC3*, which is located in the endoplasmic reticulum and catalyzes the dephosphorylation of glucose-6-phosphate, have recently been identified as resulting in autosomal recessive SCN with congenital heart disease. Patients with the *G6PC3* mutation lack mature neutrophils in the bone marrow, and their primary haematopoietic cells and fibroblasts are more likely to undergo apoptosis (16). However, with the exception of severe neutropenia, P34 in our study did not show any additional organ involvement, such as cardiac and urogenital malformations and increased venous marking. The other 32 patients had no identified gene mutations. Their persistent low neutrophil counts may be attributed to other unknown mechanism. Thus, they were designated as having SN with unknown etiology.

After excluding common secondary factors, SN over 6 months may be caused by uncommon virus infection or other etiology in some patients. Our results show that 52.63% of patients with SN with unknown etiology may maintain ANC values over 1 × 10^9^/L for >3 months after neutropenia for 19 months. Some may recover after up to 2 years, possibly due to resolution of the underlying process. Long-term SN with unknown etiology may originate from persistent unclear virus infection that cannot be easily be detected by current available laboratory techniques.

In the current study, we tried to identify distinguishing characteristics of patients with SCN, SN with unknown etiology, and recovered SN. However, all patients had early onset, similar age of diagnosis, and diagnosis lag. Consequently, pediatricians may not be able to distinguish SCN and SN with unknown etiology based on clinical data.

Early bacterial infections such as omphalitis, pneumonia, skin abscesses, and otitis media are common initial presentations of SCN (17). All of these are presented by our patients, particularly bacterial pneumonia and purulent infection. SCN patients had much more severe infections than SN with unknown etiology and recovered SN patients. Clinical presentation of SN with unknown etiology can range from asymptomatic to mild recurrent infections, which is also consistent with reports on idiopathic neutropenia (18, 19). Distinctions in the degree of infection may suggest differences in pathogenesis between SCN and SN with unknown etiology patients. Nevertheless, the possibility of recovery of patients with SN with unknown etiology cannot be predicted by clinical symptoms alone.

Median ANC and WBC counts in the patients in our study were similar to those in Razaei's study (*P* = 0.52, 0.29). However, the median monocyte count in our study was much lower (*P* < 0.001) (14). Of note, ANC and absolute monocyte counts in SCN were relatively higher, which can possibly be used to distinguish SCN patients from SN with unknown etiology patients in terms of hematological characteristics. Patients with relatively higher ANC and monocyte counts are more likely to have gene mutations and are therefore more likely to be diagnosed as having SCN. Given that there were no significant differences in ANC, monocyte and WBC counts between SN with unknown etiology and recovered SN patients, these groups cannot be distinguished based on their hematological characteristics.

The difference in the bone marrow biopsy results between SN with unknown etiology patients and SCN patients indicates the possibility that these two groups of patients undergo differential pathogenesis. An early-stage maturation arrest of myeloid differentiation was found in bone marrow studies of SCN (14). SN with unknown etiology in patients with normal nucleated cells, erythroid cells, granulocyte, and megakaryocytes may have other underlying features that affect the survival of neutrophils. Distinguishing clinical features, hematological characteristics and bone marrow characteristics between patients with SCN and SN with unknown etiology may help physicians to discriminate SCN from SN. Furthermore, such differences may imply differential pathogenesis in these 2 groups of patients, although this remains to be elucidated. Data generated from this study could not distinguish between SN with unknown etiology and recovered SN patients, nor did the data help to predict the possibility of remission in patients with SN with unknown etiology. SN with unknown etiology patients may need long-term follow-up to determine their ultimate outcome.

In summary, we analyzed the clinical and laboratory findings of Chinese SN patients and classified them as having SCN, SN with unknown etiology and recovered SN according to their gene mutations and remission status. Patients with relatively more severe infection, higher ANC and monocyte counts and maturation arrest in the bone marrow are inclined to have SCN. As a result, infection conditions, routine blood tests and bone marrow examinations appear useful for predicting the possibility of gene mutation in such patients. Future studies are required to reveal the underlying differences in the pathogenesis of SN with unknown etiology and SN recovery.

## Author contributions

R-LG was responsible for patients follow-up, data collection and analysis, and article writing. JW was responsible for data collection and article revising. T-XC was responsible for project design, patients recruitment, article conception and revising.

### Conflict of interest statement

The authors declare that the research was conducted in the absence of any commercial or financial relationships that could be construed as a potential conflict of interest. The reviewer LF and handling Editor declared their shared affiliation.
