# Supplementary material for: Clinical, Laboratory, and Molecular Characteristics and Remission Status in Children With Severe Congenital and Non-congenital Neutropenia
Source: Front Pediatr. 2018 Oct 16;6:305. doi: 10.3389/fped.2018.00305 (PMC6198072; doi:10.3389/fped.2018.00305)
Supplement: Supplementary file 1 [file Table_1.DOCX]

Supplementary Table 1. Remission status of all patients with severe neutropenia

| Pts | Neutrophil count (×10^9^/L) | Recovery age (months) | Neutropenia duration (months) | Recovery duration (months) |
| --- | --- | --- | --- | --- |
| P1 | 0.50 | / | 138 | / |
| P2 | NA | NA | NA | NA |
| P3 | NA | NA | NA | NA |
| P4 | NA | NA | NA | NA |
| P5 | NA | NA | NA | NA |
| P6 | NA | NA | NA | NA |
| P7 | 2.3 | 30 | 27 | 12 |
| P8 | 1.30 | 40 | 39 | 16 |
| P9 | 0.70 | / | 57 | / |
| P10 | 0.40 | / | 32 | / |
| P11 | NA | NA | NA | NA |
| P12 | 2.30 | 20 | 12 | 5 |
| P13 | 1.70 | 12 | 10 | 19 |
| P14 | 1.50 | 41 | 35 | 19 |
| P15 | 1.20 | 18 | 10 | 17 |
| P16 | 0.70 | / | 43 | / |
| P17 | 0.30 | / | 59 | / |
| P18 | 1.30 | 36 | 17 | 12 |
| P19 | 0.50 | / | 24 | / |
| P20 | NA | NA | NA | NA |
| P21 | NA | NA | NA | NA |
| P22 | 0.20 | / | 53 | / |
| P23 | 2.30 | 11 | 11 | 5 |
| P24 | 0.70 | / | 63 | / |
| P25 | 0.50 | / | 31 | / |
| P26 | 2.10 | 11 | 11 | 6 |
| P27 | dead | dead | dead | dead |
| P28 | NA | NA | NA | NA |
| P29 | NA | NA | NA | NA |
| P30 | NA | NA | NA | NA |
| P31 | NA | NA | NA | NA |
| P32 | 1.70 | 27 | 22 | 3 |
| P33 | 0.02 | / | 25 | / |
| P34 | 0.20 | / | 38 | / |
| P35 | 0.40 | / | 25 | / |
| P36 | 0.70 | / | 13 | / |
| P37 | 0.60 | / | 24 | / |
| P38 | 0.60 | / | 14 | / |
| P39 | 0.15 | / | 15 | / |

NA means not available At the time of study and after G-CSF therapy
