# Supplementary material for: Clinical, Laboratory, and Molecular Characteristics and Remission Status in Children With Severe Congenital and Non-congenital Neutropenia
Source: Front Pediatr. 2018 Oct 16;6:305. doi: 10.3389/fped.2018.00305 (PMC6198072; doi:10.3389/fped.2018.00305)
Supplement: Supplementary file 2 [file Table_2.DOCX]

Supplementary table 2. Hematological Characteristics of Patients with SCN, unknown etiology SN and recovered SN

| Pts | WBC  (×10^9^/L) | Neutrophil count  (×10^9^/L) | Lymphocyte count  (×10^9^/L) | Monocyte count  (×10^9^/L) | Eosinophil count  (×10^9^/L) | Platelet count  (×10^9^/L) | Hb (g/L) |  |
| --- | --- | --- | --- | --- | --- | --- | --- | --- |
| P1 | | 6.69 | 0.26 | 5.62 | 0.76 | 0.04 | 497 | 102 |
| P2 | | 2.15 | 0.38 | 1.40 | 0.36 | 0.00 | 265 | 137 |
| P3 | | 4.00 | 0.40 | 2.03 | 2.89 | 0.47 | 257 | 131 |
| P4 | | 3.42 | 0.09 | 2.75 | 0.50 | 0.04 | 317 | 118 |
| P5 | | 1.31 | 0.27 | 0.78 | 0.26 | 0.00 | 202 | 92 |
| P6 | | 4.82 | 0.01 | 2.91 | 1.79 | 0.11 | 250 | 128 |
| P7 | | 8.86 | 0.20 | 7.40 | 0.60 | 0.62 | 293 | 207 |
| P8 | | 1.70 | 0.10 | 1.20 | 0.35 | 0.04 | 332 | 121 |
| P9 | | 6.57 | 0.05 | 4.86 | 1.34 | 0.30 | 460 | 109 |
| P10 | | 6.10 | 0.50 | 3.80 | 1.60 | 0.20 | 463 | 112 |
| P11 | | 6.42 | 0.33 | 5.41 | 0.48 | 0.20 | 177 | 107 |
| P12 | | 6.80 | 0.21 | 5.78 | 0.60 | 0.20 | 101 | 105 |
| P13 | | 1.11 | 0.14 | 0.87 | 0.09 | 0.01 | 238 | 97 |
| P14 | | 3.80 | 0.30 | 2.28 | 0.30 | 0.01 | 285 | 107 |
| P15 | | 5.40 | 0.20 | 4.20 | 0.70 | 0.00 | 330 | 115 |
| P16 | | 4.15 | 0.02 | 2.57 | 1.27 | 0.23 | 520 | 100 |
| P17 | | 2.30 | 0.47 | 1.09 | 0.62 | 0.11 | 256 | 115 |
| P18 | | 3.90 | 0.40 | 3.00 | 0.50 | 0.10 | 125 | 114 |
| P19 | | 3.02 | 0.11 | 2.32 | 0.52 | 0.07 | 253 | 104 |
| P20 | | 7.56 | 0.09 | 5.82 | 0.62 | 0.01 | 299 | 105 |
| P21 | | 3.27 | 0.34 | 1.11 | 0.75 | 1.04 | 292 | 95 |
| P22 | | 1.60 | 0.10 | 1.00 | 0.40 | 0.07 | 308 | 108 |
| P23 | | 4.70 | 0.50 | 3.40 | 0.60 | 0.00 | 330 | 103 |
| P24 | | 4.55 | 0.40 | 3.50 | 1.23 | 0.11 | 288 | 147 |
| P25 | | 5.30 | 0.33 | 1.51 | 2.44 | 0.68 | 284 | 123 |
| P26 | | 5.23 | 0.25 | 4.54 | NA | 0.07 | 259 | 136 |
| P27 | | 3.40 | 0.20 | 2.42 | 0.64 | 0.05 | 473 | 107 |
| P28 | | 3.17 | 0.30 | 2.80 | 0.40 | 0.00 | 212 | 139 |
| P29 | | 4.68 | 0.50 | 3.84 | 0.29 | 0.40 | 198 | 127 |
| P30 | | 6.41 | 0.36 | 5.08 | 0.50 | 0.44 | 326 | 126 |
| P31 | | 3.73 | 0.41 | 2.74 | 0.43 | 0.13 | 527 | 101 |
| P32 | | 4.10 | 0.01 | 2.44 | 1.40 | 0.23 | 230 | 125 |
| P33 | | 4.53 | 0.29 | 3.93 | 0.57 | 0.16 | 239 | 120 |
| P34 | | 3.67 | 0.40 | 2.30 | 0.80 | 0.07 | 188 | 128 |
| P35 | | 4.04 | 0.20 | 3.30 | 0.50 | 0.08 | 268 | 112 |
| P36 | | 6.78 | 0.20 | 5.60 | 0.80 | 0.19 | 375 | 113 |
| P37 | | 5.76 | 0.12 | 4.80 | 0.48 | 0.35 | 357 | 96 |
| P38 | | 7.28 | 0.47 | 3.99 | 2.36 | 0.44 | 613 | 72 |
| P39 | | 5.29 | 0.16 | 2.66 | 2.32 | 0.15 | 438 | 137 |

NA means not available

At the time of diagnosis and before G-CSF therapy

Reference values of hematological laboratory: white blood cells (WBC ×10^9^/L), 4.0–15.0×10^9^/L; lymphocytes (×10^9^/L), 1.2-3.4×10^9^/L; monocytes (×10^9^/L), 0-0.7×10^9^/L; eosinophils (×10^9^/L), 0-0.7×10^9^/L; plateletes (×10^9^/L), 100-300×10^9^/L; hemoglobin (Hb) (g/L), 110-160g/L.
